# Knotting of the guide wires: A rare complication during minimally invasive procedure on kidney-Lessons learnt

**DOI:** 10.4103/0972-9941.45209

**Published:** 2008

**Authors:** Pankaj M Joshi, Subodh R Shivde, Tushar A Dighe

**Affiliations:** Department of Urology, Deenanath Mangeshkar Hospital, Erendawane, Pune-411 004, Maharashtra, India

**Keywords:** Guide wires-knotting-kinking

## Abstract

Guide wires are frequently used in various endourologic procedures to access the upper as well as lower urinary tract. Flexible guide wires have lesser complication rate of tissue injury as compared to stiff guide wires. Flexible guide wires are however more prone to bending and kinking due to their mechanical properties. We report an unusual complication of knotting of flexible guide wires during endourologic procedure and the trick to remedy this problem. We have also discussed the structural design and mechanical properties of commonly used guide wires.

## INTRODUCTION

A guide wire is frequently used to access the upper urinary tract. It is also used as a support for ureteric catheter placement and retrograde procedures such as ureterorenoscopy.[1] A guide wire typically contains a core composed of a superelastic nitinol alloy, covered by an exterior coating of a low friction material such as polytetrafluroethylene - a hydrophilic polymer. This particular design along with a straight or angled flexible tip of the wire provides the properties necessary to negotiate a tortuous ureter path and bypass possible obstructions. The guide wires currently used have many important properties such as the ability to push (pushability), kink resistance, torqueability, and ability to bend without breaking (bendability).[2] We report a case where the guide wires underwent knotting unexpectedly during a minimally invasive renal surgery. We also discuss the strategies used to remedy such unexpected complications encountered during commonly done minimally invasive procedures with the use of guide wires.

## CASE REPORT

A 40-year-old male patient presented to us with a 2 cm left renal calculus. He underwent a percutaneous nephrolithotomy. A lower calyceal puncture was made under fluoroscopy guidance and two guide wires (Terumo™ 0.035) were inserted. The wires entered the distal ureter and their position was confirmed on image intensifier. The nephrostomy tract was dilated over one guide wire and the other was kept as a safety wire. An Amplatz sheath was positioned and nephroscope was inserted. The stone was fragmented using a pneumatic lithotriptor and cleared completely. At the end of the procedure the Amplatz sheath was removed after inserting a tube drain of 24 French. After confirming the position of the tube drain under image intensifier an attempt was made to pull the guide wires out. An unexpected resistance was encountered during the withdrawal of both the guide wires.

**Figure 1:**
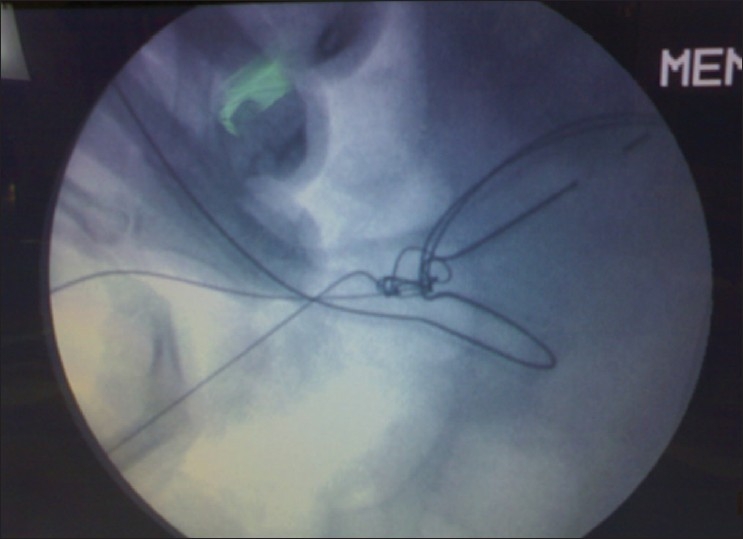
Knotting of guide wires seen on image intensifier image

On screening under image intensifier the guide wires appeared to be knotted around the tube drain - an unusual complication [Figure 1].

**Figure 2:**
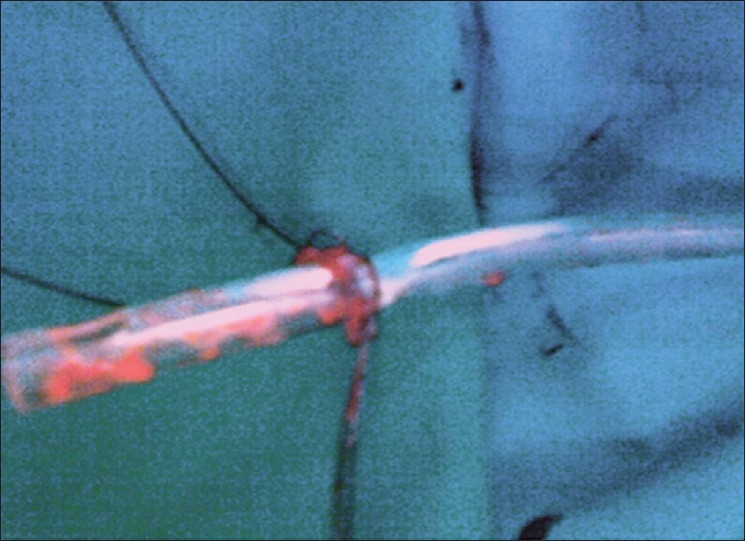
Photograph showing the knotting of guide wires over the tube drain which was removed

Several attempts were made to pull the entangled wires but they resulted in further tightening of the knot. A decision was made to remove the entire assembly of the tube drain with the knotted guidewires. This would have led to the loss of access to the pelvicalyceal system. Hence the Amplatz sheath was reinserted over the tube drain. The tube drain along with the knotted guide wires was pulled out of the pelvicalyceal system [Figure 2]. The Amplatz sheath maintained the access to the pelvicalyceal system and a new drain was reinserted to conclude the procedure successfully.

## DISCUSSION

Guide wires differ in their physical properties depending on the material they are made of. Buckling, kinking, and knotting is seen more commonly with flexible tip guide wires as compared to stiff guide wires. The stiff guide wires however have more potential for causing complications such as submucosal undermining and perforation.

Floppy tip nitinol based guide wires with a low friction coating appears to be the best for access. The stiffer shaft guide wires are reserved for co-axial passage of ureteric catheters, stents, and sheaths.[3]

Bendability is the characteristic balance between adequate flexibility to navigate tortuous lumen and suitable rigidity to support tracking of another device such as a catheter.[1] On the other hand, kink resistance is also an important characteristic of the guide wires and is closely related to the stiffness of the guide wire. Very stiff guide wires provide good pushability and are less prone to bending.[2] Variability in these properties of different guide wires cause bending, kinking, buckling, and knotting. We have encountered false passages and bending of the guide wires during endourologic procedures. Although knotting of the guide wires during minimally invasive procedures may be encountered in a busy unit the removal of the wires en-bloc may prevent the entry of endoscope back in the pelvicalyceal system. This would lead to abandonment of the procedure and may lead to complications such as failure to drain the urine and blood from the system. In our case the knotting of the guide wires around the drain needed the entire assembly consisting of tube drain and knotted wires to be pulled out en-bloc.

Knotting of the guide wires is described in literature during the subclavian vein canulation and occasionally femoral vein canulation.[5] Knotting of the urinary catheters have also been described.[6^,7^] An extensive search done on endourologic procedures in Pubmed with the key words of these problems did not reveal any such reports related to knotting of the guidewires or the plans to counter these problems. Having done a literature review and the study of physical properties of the guide wires we feel that two factors could be responsible for knotting of the guide wires:

1. Excessive length of the guide wires inserted inside a small closed space such as the pelvicalyceal system.[6^,7^]
2. The force gets transferred on to the coaxial guide wire during the insertion of nephroscope in to the Amplatz sheath. This action done repeatedly causes the coaxial wire to buckle and kink.[5] Excessive torsional force created on the access wire causes such a wire to get entangled with the stable safety guide wire.

The possible ways of avoiding such complications would be:

1. Insertion of appropriate length of the guide wire inside the pelvicalyceal system.
2. The use of stiffer guidewires instead of flexible guide wires to reduce kinking, bending, and buckling of the wires.
3. Gentle movements of the nephroscope.
